# Cardioprotective Properties of Mannitol—Involvement of Mitochondrial Potassium Channels

**DOI:** 10.3390/ijms22052395

**Published:** 2021-02-27

**Authors:** Katharina Feige, Janine Rubbert, Annika Raupach, Martin Stroethoff, André Heinen, Markus W. Hollmann, Ragnar Huhn, Carolin Torregroza

**Affiliations:** 1 Department of Anesthesiology, Medical Faculty and University Hospital Duesseldorf, Heinrich-Heine-University Duesseldorf, Moorenstr. 5, 40225 Duesseldorf, Germany; KatharinaKristina.Feige@med.uni-duesseldorf.de (K.F.); Janine.Rubbert@hhu.de (J.R.); Annika.Raupach@med.uni-duesseldorf.de (A.R.); Martin.Stroethoff@med.uni-duesseldorf.de (M.S.); Carolin.Torregroza@med.uni-duesseldorf.de (C.T.); 2 Institute of Cardiovascular Physiology, Medical Faculty and University Hospital Duesseldorf, Heinrich-Heine-University Duesseldorf, Universitaetsstr. 1, 40225 Duesseldorf, Germany; Andre.Heinen@uni-duesseldorf.de; 3 Department of Anesthesiology, Amsterdam University Medical Center (AUMC), Location AMC, Meiberdreef 9, 1105 AZ Amsterdam, The Netherlands; m.w.hollmann@amsterdamumc.nl

**Keywords:** mannitol, osmolarity, myocardial infarction, preconditioning, postconditioning

## Abstract

Cardiac preconditioning (PC) and postconditioning (PoC) are powerful measures against the consequences of myocardial ischemia and reperfusion (I/R) injury. Mannitol—a hyperosmolar solution—is clinically used for treatment of intracranial and intraocular pressure or promotion of diuresis in renal failure. Next to these clinical indications, different organ-protective properties—e.g., perioperative neuroprotection—are described. However, whether Mannitol also confers cardioprotection via a pre- and/or postconditioning stimulus, possibly reducing consequences of I/R injury, remains to be seen. Therefore, in the present study we investigated whether (1) Mannitol-induced pre- and/or postconditioning induces myocardial infarct size reduction and (2) activation of mitochondrial ATP-sensitive potassium (mK_ATP_) channels is involved in cardioprotection by Mannitol. Experiments were performed on isolated hearts of male Wistar rats via a pressure controlled Langendorff system, randomized into 7 groups. Each heart underwent 33 min of global ischemia and 60 min of reperfusion. Control hearts (Con) received Krebs–Henseleit buffer as vehicle only. Pre- and postconditioning was achieved by administration of 11 mmol/L Mannitol for 10 min before ischemia (Man-PC) or immediately at the onset of reperfusion (Man-PoC), respectively. In further groups, the mK_ATP_ channel blocker 5HD, was applied with and without Mannitol, to determine the potential underlying cardioprotective mechanisms. Primary endpoint was infarct size, determined by triphenyltetrazolium chloride staining. Mannitol significantly reduced infarct size both as a pre- (Man-PC) and postconditioning (Man-PoC) stimulus compared to control hearts (Man-PC: 31 ± 4%; Man-PoC: 35 ± 6%, each *p* < 0.05 vs. Con: 57 ± 9%). The mK_ATP_ channel inhibitor completely abrogated the cardioprotective effect of Mannitol-induced pre- (5HD-PC-Man-PC: 59 ± 8%, *p* < 0.05 vs. Man-PC) and postconditioning (5HD-PoC-Man-PoC: 59 ± 10% vs. *p* < 0.05 Man-PoC). Infarct size was not influenced by 5HD itself (5HD-PC: 60 ± 14%; 5HD-PoC: 54 ± 14%, each ns vs. Con). This study demonstrates that Mannitol (1) induces myocardial pre- and postconditioning and (2) confers cardioprotection via activation of mK_ATP_ channels.

## 1. Introduction

Ischemic conditioning still remains a strong measure to confer cardioprotection by inducing infarct size reduction and thereby protecting the heart against the detrimental consequences of ischemia and reperfusion (I/R) injury [1]. However, due to its immense invasiveness in the majority of cases, it is unattainable in daily clinical routine. For this reason, pharmacological preconditioning (PC) and postconditioning (PoC) induced by treatment with various different substances, e.g., Dexmedetomidine, volatile anesthetics or opioids, gained importance over the years and have revealed persuading results comparable to ischemic conditioning [2,3].

Mannitol, 6-carbon natural alditol, is a hyperosmolar solution and osmotherapeutic agent only scarcely metabolized and mainly excreted rapidly via the kidneys [4]. To this day, it is widely used in many fields of medicine due to its beneficial effects on the kidney [5], brain [6,7,8] and heart [4,9]. One main clinical indication is treatment of elevated intracranial pressure (ICP), as the osmotherapeutic agent is thought to decrease brain volume by reducing overall water content and thus blood volume by its osmotic and vasoconstrictive effect [9]. Further, Mannitol may also improve cerebral perfusion by decreasing viscosity or altering red blood cell rheology [9]. Next to its favorable effects on cerebral and renal function, Mannitol has been extensively used in cardiac surgery achieving an increased coronary blood flow, cardiac output, mean systemic arterial pressure, left-ventricular end-diastolic pressure and myocardial left-ventricular contractility [4]. Previous experimental studies have shown cardioprotective properties of Mannitol, mostly ascribed to its hyperosmolar or radical scavenging characteristics [10,11,12,13]. However, to this point profound data on mechanisms of Mannitol-induced cardioprotection is lacking.

The mitochondrion is not only an integral player but possibly the end effector of cardioprotection [14]. Regulation of mitochondrial function, alongside mitophagy, is crucial for cell survival after myocardial I/R injury [15,16]. Mitochondrial potassium (mK^+^) channels have been proven to play a central role in various kinds of ischemic and pharmacological conditioning strategies [17,18]. The mitochondrial ATP-sensitive potassium (mK_ATP_) channels are crucially involved in the regulation of cellular energy levels as well as organelle volume and function [19,20,21,22]. Treatment with diazoxide, a specific mK_ATP_ opener, has led to a significant infarct size reduction in experimental studies, while the respective channel blocker 5-hydroxydecanoate (5HD) averts this effect. We and others have previously demonstrated that several different pharmacological conditioning strategies are mediated via mK_ATP_, as combining 5HD with these stimuli completely abrogates cardioprotection [3].

Considering that Mannitol is already approved safe for clinical use and also employed in different perioperative settings, it appears as a promising candidate for a pharmacological conditioning strategy in patients. Given its potential clinical impact, more profound data on pre- and postconditioning as well as the underlying mechanisms of cardioprotection by Mannitol is of crucial importance. Therefore, we set out to determine whether (1) Mannitol induces cardioprotection by pre- and/or postconditioning and (2) this effect is mediated via activation of mK_ATP_ channels.

## 2. Results

### 2.1. Animal Characteristics

There were no differences detected between and within all groups of this study regarding body weight, wet and dry heart weight and level or time of maximal ischemic contracture (Table 1 and Table 2).

### 2.2. Infarct Size

**Figure 1:**
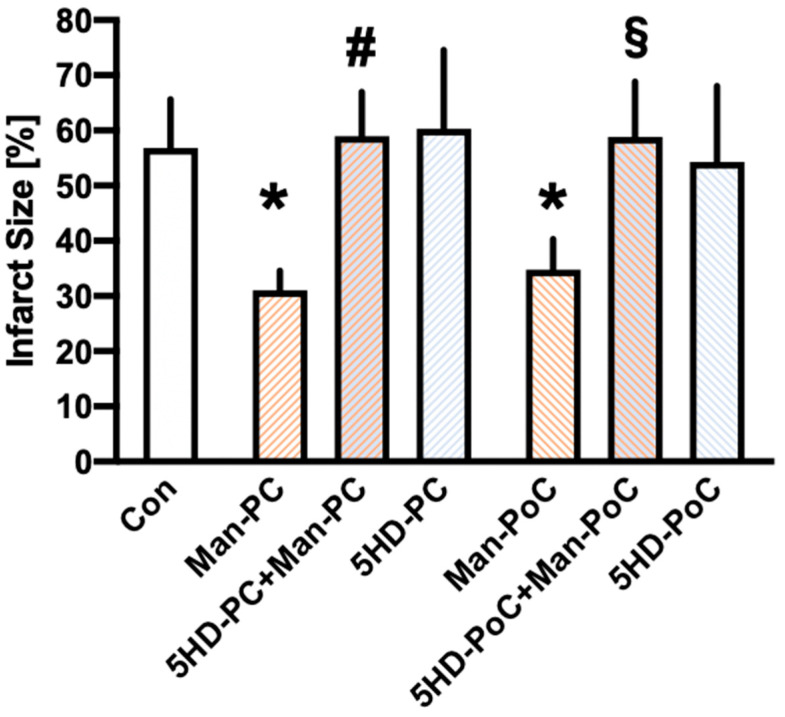
Infarct size measurement. Histogram shows all infarct sizes of the study. Data are presented as means ± SD; * *p* < 0.05 vs. Con; # *p* < 0.05 vs. Man-PC; § *p* < 0.05 vs. Man-PoC.

All infarct sizes are displayed in Figure 1. Control hearts showed an infarct size of 57 ± 9% of the left ventricle. Both pre- and postconditioning with Mannitol significantly reduced infarct size to 31 ± 4% (Man-PC, *p* < 0.05 vs. Con) and 35 ± 6% (Man-PoC, *p* < 0.05 vs. Con), respectively. The mK_ATP_ channel blocker 5HD completely abolished cardioprotection by Mannitol-induced pre- (5HD-PC+Man-PC: 59 ± 8%, *p* < 0.05 vs. Man-PC) and postconditioning (5HD-PoC+Man-PoC: 59 ± 10%, *p* < 0.05 vs. Man-PoC). The channel blocker itself had no effect on infarct size (5HD-PC: 60 ± 14%, ns vs. Con and 5HD-PoC: 54 ± 14%, ns vs. Con).

### 2.3. Cardiac Function

Hemodynamic variables are shown in Table 3. There were no differences detected between the different groups for each time of measurement. Heart rate slightly decreased from baseline to the end of reperfusion, but no significant differences were measured within or between groups. Both left ventricular-developed pressure and coronary flow significantly decreased during reperfusion compared to baseline values within each study group.

## 3. Discussion

In this study, we demonstrated infarct size-reducing effects by Mannitol-induced pre- and postconditioning and reported novel possible underlying mechanisms of cardioprotection by Mannitol.

Cardioprotective properties of Mannitol have been described over several years in various animal species and experimental setups and were mostly ascribed to its hyperosmolar characteristics or possible radical scavenging features [12,23,24]. Magovern et al. showed that reperfusion with Mannitol resulted in improved developed pressure, greater coronary flow and less myocardial edema due to its osmotherapeutic effects [12]. However, studies on cardioprotection and hyperosmolarity show inconsistent results. Intracoronary administration of Mannitol in different in vivo experiments for instance did not improve heart resistance against I/R injury [25,26]. Hence, Mannitol-induced cardioprotection might not only be explained by its osmotic characteristics.

Next to several known signaling cascades, oxidative stress by reactive oxygen species (ROS) has been discussed as playing a key role in I/R injury [3,18,27]. Even though low levels of ROS are needed for cardioprotection, excessive ROS formation ultimately leads to cell death after I/R injury. As Mannitol is known to be an effective free radical scavenger, it appears likely that the osmotherapeutic agent might confer its organ-protective properties via regulation of ROS levels during ischemia and reperfusion. While Ouriel et al. [24] suggested that Mannitol offers myocardial protection not only via hyperosmolarity but also possibly due to free radical scavenging activity, an ex vivo study by Pastukh et al. [28] did not support this concept of cardioprotection by Mannitol. In isolated cardiomyocytes, pretreatment with Mannitol showed no effect on the generation of free radicals during hypoxia [28]. Yet, hyperosmolarity by Mannitol induced activation of phosphoinositide 3-kinase (PI 3K)/protein kinase B (Akt) pathway contributing to cardioprotection. Gardner et al. [11] underline these findings by investigating the effect of Mannitol on the oxygen paradox in an in vitro I/R injury study. Results indicate that Mannitol reduces reoxygenation-induced cardiac biomarker release by osmotic activity, rather than by its oxygen-free radical scavenger abilities. Hence, to this date clear consensus on the underlying mechanisms of Mannitol-induced myocardial protection has been lacking. Moreover, studies on Mannitol and cardioprotection used different conditioning strategies, time or duration of application as well as varying substance concentrations. For instance, preconditioning with Mannitol was demonstrated by Falck et al. [10] through perfusion with the hyperosmotic (600 mOsm/kg) solution before ischemia and reperfusion. However, only a 2 min treatment with Mannitol induced infarct size reduction, while 1 min or even 5 min of application showed no effect. Thus, next to a lack of profound knowledge on underlying mechanisms of cardioprotection by Mannitol, studies investigating different conditioning stimuli with clinically used concentrations have also been sparse to this point.

Various intracellular signaling pathways are known to play a crucial role in cardioprotection, and it is accepted that several mediators finally converge upon and regulate the mitochondria ultimately providing protection against myocardial I/R injury [14,15,27]. Next to different intracellular mediators, mitochondrial potassium channels have been identified as integral players in cardiac conditioning by several different ischemic and pharmacological stimuli [17,19,29,30]. These channels are closely linked to the modulation of mitochondrial function, especially considering the energetic status, electrolyte influx and, hence, membrane potential [31]. Activation of these mK^+^ channels is generally considered to inhibit the mitochondrial permeability transition pore (mPTP). Prolonged opening of the mPTP leads to mitochondrial swelling, release of pro-apoptotic factors and ultimately cell death after I/R injury. Thus, inhibition of mPTP opening is a key step in myocardial protection [32,33].

Our findings demonstrate for the first time, that mK_ATP_ are involved in the cardioprotective effect by Mannitol, as the mK_ATP_ channel inhibitor 5HD completely abolished infarct size reduction by Mannitol. A potent and selective blocker of the mK_ATP_ channel [34], 5HD has been widely used in several different experimental studies successfully blocking the respective channel [35,36,37,38]. In more detail, 5HD mimics ATP by binding to the mitochondrial potassium channel. With regard to its effectiveness, it was recently reported that 5HD in a concentration of 100 µM—the same concentration used in our experiments—leads to a complete blockage of the mK_ATP_ channel [21]. While results from our study support that pre- and postconditioning with Mannitol leads to cardioprotection via mK_ATP_ channel activation, at this point it still remains to be discussed how exactly Mannitol confers the opening of these respective channels. There are different signaling pathways, e.g., reperfusion injury salvage kinase (RISK), survivor activating factor enhancement (SAFE) and nitric oxide/protein kinase G (NO/PKG), recognized to target the mitochondrion as an end-effector of cardioprotection after I/R injury. Referring to mK_ATP_ channels, both protein kinase C (PKC) as part of the RISK pathway and NO/PKG have been shown to take a central role in intracellular signaling. These signaling cascades and downstream targets are for instance activated by the binding of specific agonists to G-protein coupled receptors located at the plasma membrane [30,39,40]. It could be assumed that Mannitol might influence these pathways, ultimately targeting the mK_ATP_ channels; however, further research is needed to fully unravel the involved mechanisms. Despite knowledge on these respective pathways involved in mK_ATP_ channel activation and cardioprotection, an osmotic effect of Mannitol or the channel itself cannot be fully excluded. There is experimental evidence that ischemia (especially during early phases) leads to matrix contracture caused by a lack of oxygen, depolarization of mitochondrial membrane and, hence, decrease of K^+^ influx [39]. In contrast, activation of the mK_ATP_ channel leads to moderate matrix swelling via osmotic effects preserving intermembrane space and in turn—via a complex interaction of different intermediates—providing adequate ATP support upon reperfusion, counteracting the negative consequences of ischemia. Referring to the possible osmotic action of Mannitol, we further looked at overall edema or swelling within the different study groups. In the experimental setup, all hearts fulfilled the same basic prerequisite, permitting a comparison of the wet and dry heart weight at the end of the experiment (see Table 1). In line with findings from Carlson et al. and Klein et al. regarding Mannitol and hyperosmolarity [25,26], there were no significant differences in wet heart weight in control hearts (perfused with Krebs–Henseleit buffer) compared to hearts perfused with Mannitol as a pre- or postconditioning stimulus. At the end of reperfusion, all hearts were weighed and dried before reweighing. From this, a determination of the content of water as a percentage can be differentiated (weight wet−weight dry/weight wet × 100). Focusing on control hearts and those treated with Mannitol solely, the water percentage results do not show a significant difference between groups. This indicates that Mannitol-induced cardioprotection might not only be explained by its hyperosmolar characteristics resulting in a reduction of edema. However, both edema and cardiac dysfunction have a number of promotors, making a direct causal relationship difficult to prove.

Although we see a strong effect on infarct size reduction by both Mannitol-induced pre- and postconditioning, no hemodynamic improvement during the reperfusion phase was detected between groups. The exact reason for this remains unknown, however the occurrence of myocardial stunning after global ischemia is often discussed in this context, meaning a temporary depression of function in the surviving myocardial tissue, especially after global ischemia. As the global function of the left ventricle is measured, we cannot discriminate between effects that belong to differences in infarct size or differences in the degree of stunning [41].

The respective Mannitol concentration of 11 mmol/L employed in this study was taken from the literature as well as one of our previous studies. In a preliminary experimental protocol, we investigated the influence of hyperglycemia on cardioprotection by Dexmedetomidine [42]. To ensure comparable osmolarity in all experiments, normoglycemic groups were treated with Mannitol—achieving the same osmolar conditions to hyperglycemia without affecting glucose levels. Interestingly, our results showed that 11 mmol/L Mannitol—administered throughout the whole experimental period— induced a strong cardioprotective effect, significantly reducing the infarct size. Along with our own findings, the respective concentration has been used in different experimental studies investigating organ-protective effects of the osmotherapeutic agent. Zálešák et al. demonstrated similar results to our previous own study, where administration of 11 mmol/L Mannitol over a prolonged experimental period induced a significant infarct size reduction [13]. They also detected a suppressed release of heart fatty acid binding protein (h-FABP) as a biomarker of myocardial cell injury. Regarding translation into the clinical setting, the employed Mannitol concentration in our in vitro study converts to around 1 g/kg bodyweight, which is exactly in line with the routinely used clinical dosage.

The present study presents Mannitol as a pre- and postconditioning stimulus in a clinically used concentration effectively reducing infarct size after I/R injury. Furthermore, we report mK_ATP_ channel activation as an alternative and novel underlying mechanism of Mannitol-induced cardioprotection, independent of the known hyperosmolar or radical scavenger characteristics.

## 4. Materials and Methods

The present investigation is in accordance with the Guide for the Care and Use of Laboratory Animals published by the U.S. National Institute of Health (NIH publication No.85-23, revised 1996) and was conducted after obtaining approval by the local Animal Care and Use Committee of the University of Duesseldorf (project number O27/12). The animals were provided from the breeding facility at the Central Animal Research Facility of Heinrich Heine University Duesseldorf.

### 4.1. Surgical Preparation

All experiments were performed on male Wistar rats (2–3 months old), as described previously [42]. In short, animals were anesthetized with intraperitoneal injection of pentobarbital (80 mg/kg body weight, Narcoren, Merial, Germany) and decapitated. A thoracotomy was performed to excise the hearts, which were then mounted onto a Langendorff system perfused with Krebs–Henseleit buffer (118 mM NaCl, 4.7 mM KCl, 1.2 mM MgSO_4_, 1.17 mM KH_2_PO_4_, 24.9 mM NaHCO_3_, 2.52 mM CaCl_2_, 11 mM glucose and 1mM lactate) under constant pressure (80 mmHg) and temperature (37 °C). The Krebs–Henseleit buffer was supplemented with a mixture of 95% O_2_ and 5% CO_2_. For implementing continuous pressure measurements, a saline-filled balloon was inserted into the left ventricle and the end-diastolic pressure was adjusted to 4–6 mmHg. The continuous measurement of the hemodynamic data (including heart rate, left ventricular end-systolic pressure (LVESP), left ventricular end-diastolic pressure (LVEDP) and coronary flow) was attained by using an analogue-to-digital converter (PowerLab/8SP, ADInstruments Pty Ltd., Castle Hill, Australia) at a sampling rate of 500 Hz. Data were recorded using Labchart 8.0 for Windows (ADInstruments Pty Ltd., Castle Hill, Australia). Left ventricular-developed pressure (LVDP) was calculated as LVESP—LVEDP. Additionally, maximal contracture during ischemia and the respective timepoint was analyzed for each experiment as an indicator for myocardial injury. At the end of reperfusion, hearts were removed from the Langendorff system, cut into 8 transverse slices (2 mm each) and stained with 0.75% triphenyltetrazolium chloride (TTC) solution. The size of the infarcted area was determined by planimetry using SigmaScan Pro5 software by a blinded, experienced investigator [43]. Infarct size was determined as percentage of infarct area per total area of the left ventricle.

### 4.2. Experimental Protocol

**Figure 2:**
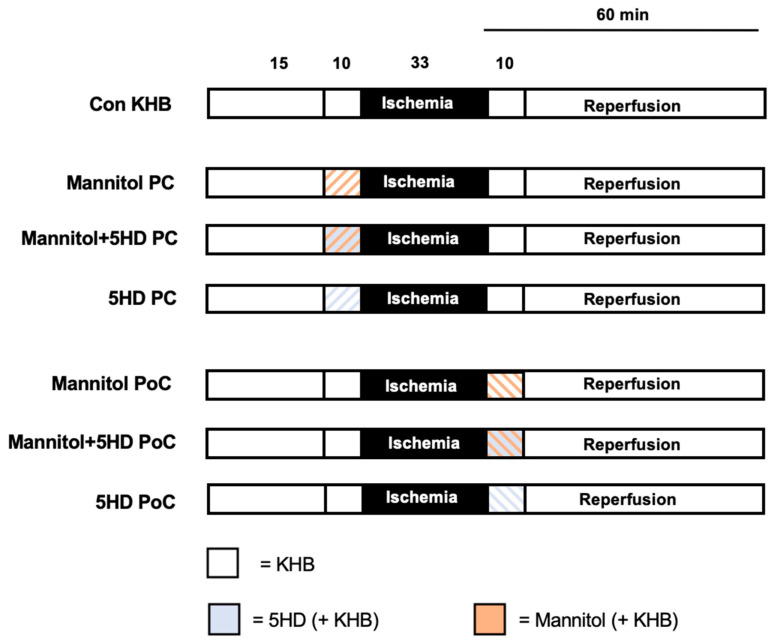
Experimental protocol. Con = Control; KHB = Krebs–Henseleit buffer; 5HD = 5-hydroxy-decanoate; PC = Preconditioning; PoC = Postconditioning.

Hearts were randomized into 7 experimental groups (*n* = 6–7 per group) as shown in Figure 2. All hearts underwent a 15 min adaption period, a 10 min preconditioning period and 33 min of ischemia, followed by 60 min of reperfusion including a 10 min postconditioning period. Global ischemia was achieved by complete cessation of retrograde perfusion. At the end of ischemia, perfusion was restored to initiate the reperfusion period.

Mannitol was applied in a concentration of 11 mmol/L as a preconditioning stimulus (PC) before or a postconditioning stimulus (PoC) after ischemia. This concentration of Mannitol was determined to induce significant infarct size reduction when applied permanently—throughout the entire experiment—in a previously performed study [42].

In further groups, Mannitol and 5HD were applied simultaneously as either a preconditioning stimulus (PC) or postconditioning stimulus (PoC). Additionally, 5HD was also given individually to rule out a possible effect of the channel blocker itself on infarct size. The concentration of 5HD was taken from the literature [21]. All substances were applied at an infusion rate of 1% of coronary flow.

Control (Con KHB): Hearts received Krebs–Henseleit buffer (KHB) as vehicle during both treatment phases (pre- and postconditioning).

Man PC: Hearts were perfused with 11 mmol/L Mannitol for 10 min before ischemia.

Man+5HD-PC: Hearts were treated with 11 mmol/L Mannitol and 100 μM 5HD for 10 min before ischemia.

5HD-PC: Hearts were treated solely with 100 μM 5HD for 10 min before ischemia.

Man PoC: Hearts were perfused with 11 mmol/L Mannitol for 10 min at the onset of reperfusion.

Man+5HD-PoC: Hearts were treated with 11 mmol/L Mannitol and 100 μM 5HD for 10 min at the onset of reperfusion.

5HD-PoC: Hearts were treated solely with 100 μM 5HD for 10 min at the onset of reperfusion.

### 4.3. Statistical Analysis

#### 4.3.1. Sample Size Analysis

We performed a sample size calculation (GraphPad StatMate™, GraphPad Software, San Diego, CA, USA) revealing a group size of *n* = 7 for detecting a 25% mean difference and a standard deviation of 15% in infarct size (power 80%, α < 0.05 (two-tailed)).

#### 4.3.2. Statistical Approach

A two-way analysis of variance (ANOVA) and a Tukey’s post hoc test (GraphPad Software V7.01, San Diego, CA, USA) were employed for comparison of hemodynamic data between groups as well as between different timepoints within groups. All data is presented as mean ± standard deviation (SD). Infarct sizes were analyzed by a one-way ANOVA and a Tukey’s post hoc test. *p* < 0.05 was considered statistically significant for changes within and between groups.

## 5. Conclusions

The results in our study demonstrate that administration of Mannitol in a clinically used concentration as pre- and postconditioning confers cardioprotection after I/R injury. Furthermore, this effect is mediated via activation of mK_ATP_ channels. These findings might be of high clinical relevance, as Mannitol is already routinely used in patients. Considering this fact, we employed a clinically used concentration in our experimental study; our results indicate that Mannitol might be a promising agent for establishing cardioprotection in the clinical setting.

## Figures and Tables

**Table 1:** Body weights and wet and dry heart weights.

	*n*	Body Weight (g)	Dry Heart Weight (g)	Wet Heart Weight (g)
Con	7	292 ± 11	0.11 ± 0.01	1.15 ± 0.05
Man-PC	6	295 ± 22	0.12 ± 0.02	1.16 ± 0.19
5HD-PC+Man-PC	7	297 ± 14	0.11 ± 0.01	1.25 ± 0.10
5HD-PC	6	297 ± 12	0.10 ± 0.02	1.18 ± 0.09
Man-PoC	6	290 ± 14	0.10 ± 0.01	1.17 ± 0.17
5HD-PoC+Man-PoC	7	302 ± 20	0.09 ± 0.01	1.16 ± 0.11
5HD-PoC	6	288 ± 10	0.11 ± 0.01	1.12 ± 0.07

Data are mean ± SD. Con = Control; Man = Mannitol; 5HD = 5-hydroxydecanoate; PC = Preconditioning; PoC = Postconditioning.

**Table 2:** Time and level of maximum ischemic contracture.

	*n*	Time of Max. Ischemic Contracture (min)	Level of Max. Ischemic Contracture (mmHg)
Con	7	14 ± 2	66 ± 8
Man-PC	6	14 ± 1	61 ± 7
5HD-PC+Man-PC	7	15 ± 2	58 ± 14
5HD-PC	6	15 ± 1	60 ± 9
Man-PoC	6	15 ± 1	64 ± 11
5HD-PoC+Man-PoC	7	15 ± 2	80 ± 9
5HD-PoC	6	15 ± 1	65 ± 6

Data are mean ± SD. Con = Control; Man = Mannitol; 5HD = 5-hydroxydecanoate; PC = Preconditioning; PoC = Postconditioning.

**Table 3:** Hemodynamic variables.

	Baseline	PC	Reperfusion	
			30	60
*Heart Rate* (bpm)				
Con	318 ± 40	318 ± 51	338 ± 75	277 ± 62
Man-PC	293 ± 20	284 ± 22	273 ± 56	233 ± 60
5HD-PC+Man-PC	300 ± 40	292 ± 35	291 ± 58	263 ± 69
5HD-PC	314 ± 46	329 ± 52	275 ± 46	227 ± 57
Man-PoC	312 ± 38	306 ± 21	253 ± 51	266 ± 17
5HD-PoC+Man-PoC	301 ± 49	289 ± 45	288 ± 63	270 ± 67
5HD-PoC	297 ± 30	288 ± 18	286 ± 29	242 ± 37
*Left Ventricular-Developed Pressure* (mmHg)				
Con	118 ± 24	122 ± 22	12 ± 6 *	24 ± 9 *
Man-PC	114 ± 9	111 ± 23	21 ± 14 *	33 ± 12 *
5HD-PC+Man-PC	112 ± 12	117 ± 9	21 ± 13 *	29 ± 14 *
5HD-PC	114 ± 10	121 ± 16	14 ± 9 *	22 ± 9 *
Man-PoC	106 ± 11	119 ± 16	27 ± 14 *	32 ± 10 *
5HD-PoC+Man-PoC	135 ± 15	145 ± 15 #	18 ± 13 *	25 ± 19 *
5HD-PoC	123 ± 21	133 ± 16	22 ± 13 *	36 ± 14 *
*Left Ventricular End Diastolic Pressure* (mmHg)				
Con	3 ± 2	4 ± 3	85 ± 23 *	72 ± 21 *
Man-PC	3 ± 1	3 ± 2	83 ± 16 *	72 ± 14 *
5HD-PC+Man-PC	4 ± 2	4 ± 2	81 ± 14 *	70 ± 12 *
5HD-PC	3 ± 1	4 ± 3	77 ± 20 *	67 ± 20 *
Man-PoC	3 ± 2	3 ± 2	75 ± 14 *	65 ± 13 *
5HD-PoC+Man-PoC	5 ± 2	5 ± 2	109 ± 18 *	97 ± 17 *
5HD-PoC	5 ± 2	5 ± 2	103 ± 10 *	88 ± 7 *
*Coronary Flow* (mL/min)				
Con	14 ± 2	14 ± 2	8 ± 1 *	8 ± 2 *
Man-PC	14 ± 2	14 ± 4	8 ± 1 *	7 ± 1 *
5HD-PC+Man-PC	14 ± 3	15 ± 3	9 ± 3 *	9 ± 3 *
5HD-PC	13 ± 2	15 ± 1	6 ± 2 *	6 ± 2 *
Man-PoC	14 ± 3	14 ± 3	8 ± 2 *	7 ± 1 *
5HD-PoC+Man-PoC	14 ± 2	14 ± 2	7 ± 2 *	7 ± 2 *
5HD-PoC	14 ± 3	13 ± 3	7 ± 2 *	6 ± 2 *

Data are mean ± SD. Con = Control; Man = Mannitol; 5HD = 5-hydroxydecanoate; PC = Preconditioning; PoC = Postconditioning. * *p* < 0.05 vs. Baseline; # *p* < 0.05 vs. Con.

## Data Availability

Not applicable.

## References

[1] Hausenloy D.J., Barrabes J.A., Bøtker H.E., Davidson S.M., Di Lisa F., Downey J., Engstrom T., Ferdinandy P., Carbrera-Fuentes H.A., Heusch G. (2016). Ischaemic conditioning and targeting reperfusion injury: A 30 year voyage of discovery. Basic Res. Cardiol..

[2] Caricati-Neto A., Errante P.R., Menezes-Rodrigues F.S. (2019). Recent Advances in Pharmacological and Non-Pharmacological Strategies of Cardioprotection. Int. J. Mol. Sci..

[3] Torregroza C., Raupach A., Feige K., Weber N.C., Hollmann M.W., Huhn R. (2020). Perioperative Cardioprotection: General Mechanisms and Pharmacological Approaches. Anesth. Analg..

[4] Poullis M. (1999). Mannitol and Cardiac Surgery. Thorac. Cardiovasc. Surg..

[5] O’Kane D., Baldwin G.S., Bolton D.M., Ischia J.J., Patel O. (2019). Preconditioning against renal ischaemia reperfusion injury: The failure to translate to the clinic. J. Nephrol..

[6] Schilte C., Bouzat P., Millet A., Boucheix P., Pernet-Gallay K., Lemasson B., Barbier E.L., Payen J.-F. (2015). Mannitol Improves Brain Tissue Oxygenation in a Model of Diffuse Traumatic Brain Injury*. Crit. Care Med..

[7] Farrokh S., Cho S.-M., Suarez J.I. (2019). Fluids and hyperosmolar agents in neurocritical care. Curr. Opin. Crit. Care.

[8] Witherspoon B., Ashby N.E. (2017). The Use of Mannitol and Hypertonic Saline Therapies in Patients with Elevated Intracranial Pressure. Nurs. Clin. N. Am..

[9] Davis M., Lucatorto M. (1994). Mannitol Revisited. J. Neurosci. Nurs..

[10] Falck G., Schjott J., Jynge P. (1999). Hyperosmotic pretreatment reduces infarct size in the rat heart. Physiol. Res..

[11] Gardner T.J., Stewart J.R., Casale A.S., Downey J.M., Chambers D.E. (1983). Reduction of myocardial ischemic injury with oxygen-derived free radical scavengers. Surgery.

[12] Magovern G.J., Bolling S.F., Casale A.S., Bulkley B.H., Gardner T.J. (1984). The mechanism of mannitol in reducing ischemic injury: Hyperosmolarity or hydroxyl scavenger?. Circulation.

[13] Zálešák M., Blažíček P., Pancza D., Gablovský I., Štrbák V., Ravingerová T. (2016). Hyperosmotic Environment Blunts Effectivity of Ischemic Preconditioning Against Ischemia-Reperfusion Injury and Improves Ischemic Tolerance in Non-Preconditioned Isolated Rat Hearts. Physiol. Res..

[14] Boengler K., Lochnit G., Schulz R. (2018). Mitochondria “THE” target of myocardial conditioning. Am. J. Physiol. Circ. Physiol..

[15] Di Lisa F., Canton M., Menabò R., Kaludercic N., Bernardi P. (2007). Mitochondria and cardioprotection. Hear. Fail. Rev..

[16] Morciano G., Patergnani S., Bonora M., Pedriali G., Tarocco A., Bouhamida E., Marchi S., Ancora G., Anania G., Wieckowski M.R. (2020). Mitophagy in Cardiovascular Diseases. J. Clin. Med..

[17] Smith C.O., Nehrke K., Brookes P.S. (2017). The Slo(w) path to identifying the mitochondrial channels responsible for ischemic protection. Biochem. J..

[18] Heusch G. (2015). Molecular Basis of Cardioprotection. Circ. Res..

[19] Ardehali H., O’Rourke B. (2005). Mitochondrial K channels in cell survival and death. J. Mol. Cell. Cardiol..

[20] Gross G.J., Peart J.N. (2003). KATP channels and myocardial preconditioning: An update. Am. J. Physiol. Circ. Physiol..

[21] Paggio A., Checchetto V., Campo A., Menabò R., Di Marco G., Di Lisa F., Szabo I., Rizzuto R., De Stefani D. (2019). Identification of an ATP-sensitive potassium channel in mitochondria. Nat. Cell Biol..

[22] Queliconi B.B., Wojtovich A.P., Nadtochiy S.M., Kowaltowski A.J., Brookes P.S. (2011). Redox regulation of the mitochondrial KATP channel in cardioprotection. Biochim. Biophys. Acta (BBA) Bioenergy.

[23] Garcia-Dorado D., Oliveras J. (1993). Myocardial oedema: A preventable cause of reperfusion injury?. Cardiovasc. Res..

[24] Ouriel K., Ginsburg M.E., Patti C.S., Pearce F.J., Hicks G.L. (1985). Preservation of myocardial function with mannitol reperfusate. Circulation.

[25] Carlson R.E., Aisen A.M., Buda A.J. (1992). Effect of reduction in myocardial edema on myocardial blood flow and ventricular function after coronary reperfusion. Am. J. Physiol. Circ. Physiol..

[26] Klein H.H., Nebendahl K., Schubothe M., Kreuzer H. (1985). Intracoronary hyperosmotic mannitol during reperfusion does not affect infarct size in ischemic, reperfused porcine hearts. Basic Res. Cardiol..

[27] Perrelli M.-G. (2011). Ischemia/reperfusion injury and cardioprotective mechanisms: Role of mitochondria and reactive oxygen species. World J. Cardiol..

[28] Pastukh V., Ricci C., Solodushko V., Mozaffari M., Schaffer S.W. (2005). Contribution of the PI 3-kinase/Akt survival pathway toward osmotic preconditioning. Mol. Cell. Biochem..

[29] Cao Y., Zhang S.-Z., Zhao S.-Q., Bruce I.C. (2011). The mitochondrial Ca2+-activated K+ channel contributes to cardioprotection by limb remote ischemic preconditioning in rat. Life Sci..

[30] Vishwakarma V.K., Upadhyay P.K., Chaurasiya H.S., Srivasatav R.K., Ansari T.M., Srivastava V. (2019). Mechanistic Pathways of ATP Sensitive Potassium Channels Referring to Cardio-Protective Effects and Cellular Functions. Drug Res..

[31] Zorova L.D., Popkov V.A., Plotnikov E.Y., Silachev D.N., Pevzner I.B., Jankauskas S.S., Babenko V.A., Zorov S.D., Balakireva A.V., Juhaszova M. (2018). Mitochondrial membrane potential. Anal. Biochem..

[32] Di Lisa F., Carpi A., Giorgio V., Bernardi P. (2011). The mitochondrial permeability transition pore and cyclophilin D in cardioprotection. Biochim. Biophys. Acta (BBA) Bioenergy.

[33] Hausenloy D.J. (2003). The mitochondrial permeability transition pore: Its fundamental role in mediating cell death during ischaemia and reperfusion. J. Mol. Cell. Cardiol..

[34] Sato T., Saito T., Saegusa N., Nakaya H. (2005). Mitochondrial Ca 2+ -Activated K + Channels in Cardiac Myocytes. Circulation.

[35] Stroethoff M., Christoph I., Behmenburg F., Raupach A., Bunte S., Senpolat S., Heinen A., Hollmann M.W., Mathes A., Huhn R. (2018). Melatonin Receptor Agonist Ramelteon Reduces Ischemia-Reperfusion Injury Through Activation of Mitochondrial Potassium Channels. J. Cardiovasc. Pharmacol..

[36] Cao S., Liu Y., Wang H., Mao X., Chen J., Liu J., Xia Z., Zhang L., Liu X., Yu T. (2016). Ischemic postconditioning influences electron transport chain protein turnover in Langendorff-perfused rat hearts. PeerJ.

[37] Li W., Wu N., Shu W., Jia D., Jia P. (2015). Pharmacological preconditioning and postconditioning with nicorandil attenuates ischemia/reperfusion-induced myocardial necrosis and apoptosis in hypercholesterolemic rats. Exp. Ther. Med..

[38] Lucchinetti E., Jamnicki M., Fischer G., Zaugg M. (2008). Preconditioning by Isoflurane Retains Its Protection Against Ischemia-Reperfusion Injury in Postinfarct Remodeled Rat Hearts. Anesthesia Analg..

[39] Dos Santos P., Kowaltowski A.J., Laclau M.N., Seetharaman S., Paucek P., Boudina S., Thambo J.-B., Tariosse L., Garlid K.D. (2002). Mechanisms by which opening the mitochondrial ATP- sensitive K+ channel protects the ischemic heart. Am. J. Physiol. Circ. Physiol..

[40] Rotko D., Kunz W.S., Szewczyk A., Kulawiak B. (2020). Signaling pathways targeting mitochondrial potassium channels. Int. J. Biochem. Cell Biol..

[41] Flameng W. (1993). Mechanisms Underlying Myocardial Stunning. J. Card. Surg..

[42] Torregroza C., Feige K., Schneider L., Bunte S., Stroethoff M., Heinen A., Hollmann M.W., Huhn R., Raupach A. (2020). Influence of Hyperglycemia on Dexmedetomidine-Induced Cardioprotection in the Isolated Perfused Rat Heart. J. Clin. Med..

[43] Behmenburg F., Dorsch M., Huhn R., Mally D., Heinen A., Hollmann M.W., Berger M.M. (2015). Impact of Mitochondrial Ca2+-Sensitive Potassium (mBKCa) Channels in Sildenafil-Induced Cardioprotection in Rats. PLoS ONE.

